# Supplementary material for: Interactions with bacteria shape diatom adaptation to carbon concentration changes
Source: Nat Commun. 2025 Dec 27;17:1289. doi: 10.1038/s41467-025-68050-3 (PMC12868695; doi:10.1038/s41467-025-68050-3)
Supplement: Supplementary file 1 — Supplementary Information [file 41467_2025_68050_MOESM1_ESM.pdf]

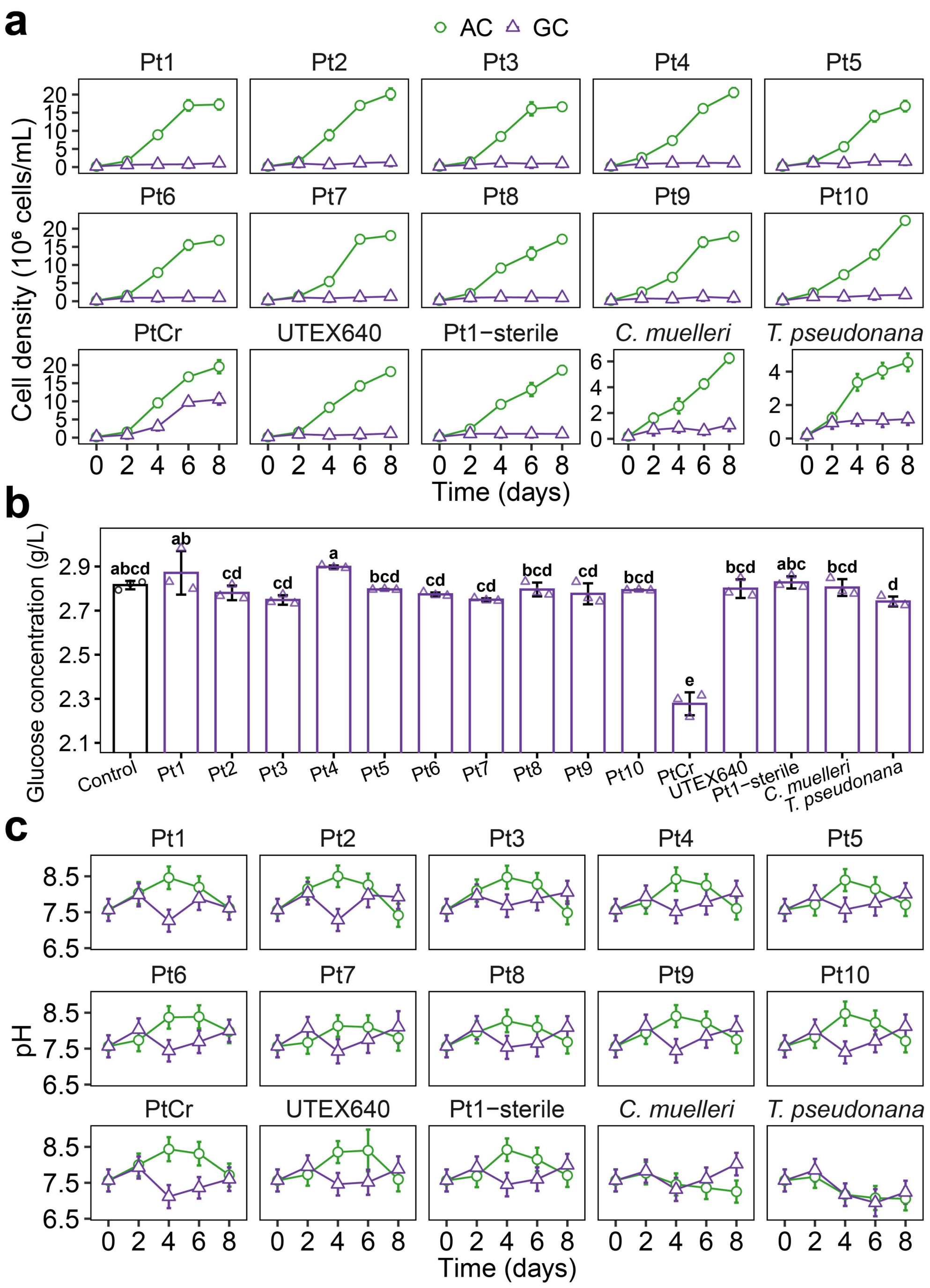

Supplementary Fig. 1 Cell density dynamics (**a**), glucose concentration under GC condition after 8 day cultivation (**b**), and pH values (**c**) of 13 *P. tricornutum* strains together with *C. muelleri* and *T. pseudonana* in cultures with supplemented silicate. The line and bar plots represent mean ± standard error (n = 3 biological replicates). Pt1-sterile, Pt1 with intensified antibiotic treatment; AC, using atmospheric CO_2 as the sole carbon source; GC, using 3 g/L glucose as the sole carbon source; Control, initial glucose concentration. Different letters above the bars indicate statistically significant differences by two-tailed Fisher's LSD test (32 degrees of freedom, $p <$ 0.05, Benjamini-Hochberg correction). Source data are provided as a Source Data file.

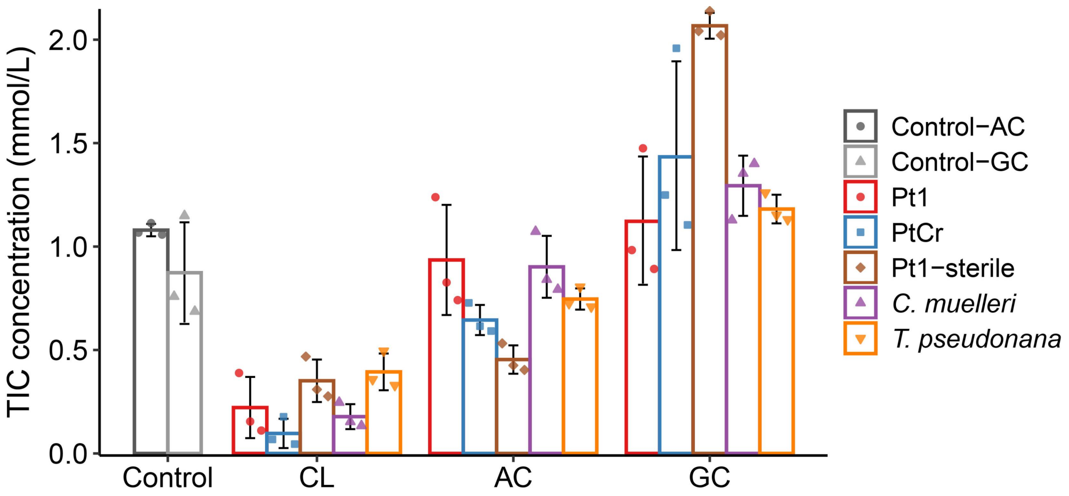

Supplementary Fig. 2 Total inorganic carbon (TIC) concentration of 3 *P. tricornutum* strains together with *C. muelleri* and *T. pseudonana* under CL, AC, and GC after 8 day cultivation with addition of silicate. The bar plots represent mean ± standard error (n = 3 biological replicates). Pt1-sterile, Pt1 with intensified antibiotic treatment; CL, carbon limitation; AC, using atmospheric CO_2 as the sole carbon source; GC, using 3 g/L glucose as the sole carbon source; Control-AC and Control-GC, initial TIC concentration under AC and GC. Source data are provided as a Source Data file.

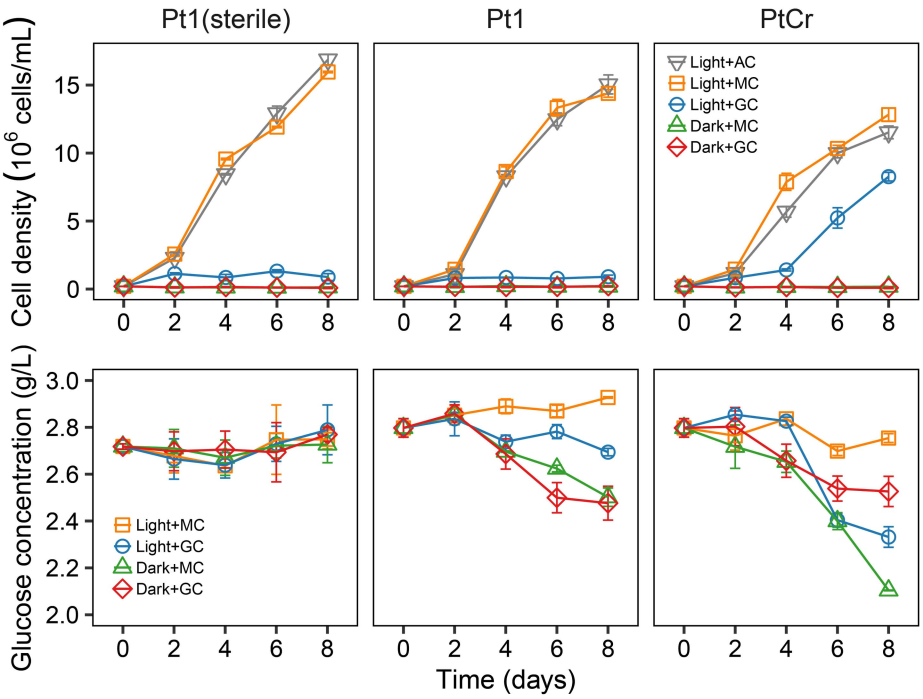

Supplementary Fig. 3 Cell density (**a**) and glucose concentration (**b**) of *P. tricornutum* (Pt1-sterile, Pt1, and PtCr) cultured under different conditions. The line plots represent mean ± standard error (n = 3 biological replicates). AC, using atmospheric CO_2 as the sole carbon source with light; MC and MC-Dark, using mixed carbon sources (atmospheric CO_2 and 3 g/L glucose) with light and in dark, respectively; GC and GC-Dark, using 3 g/L glucose as the sole carbon source with light and in dark, respectively. Source data are provided as a Source Data file.

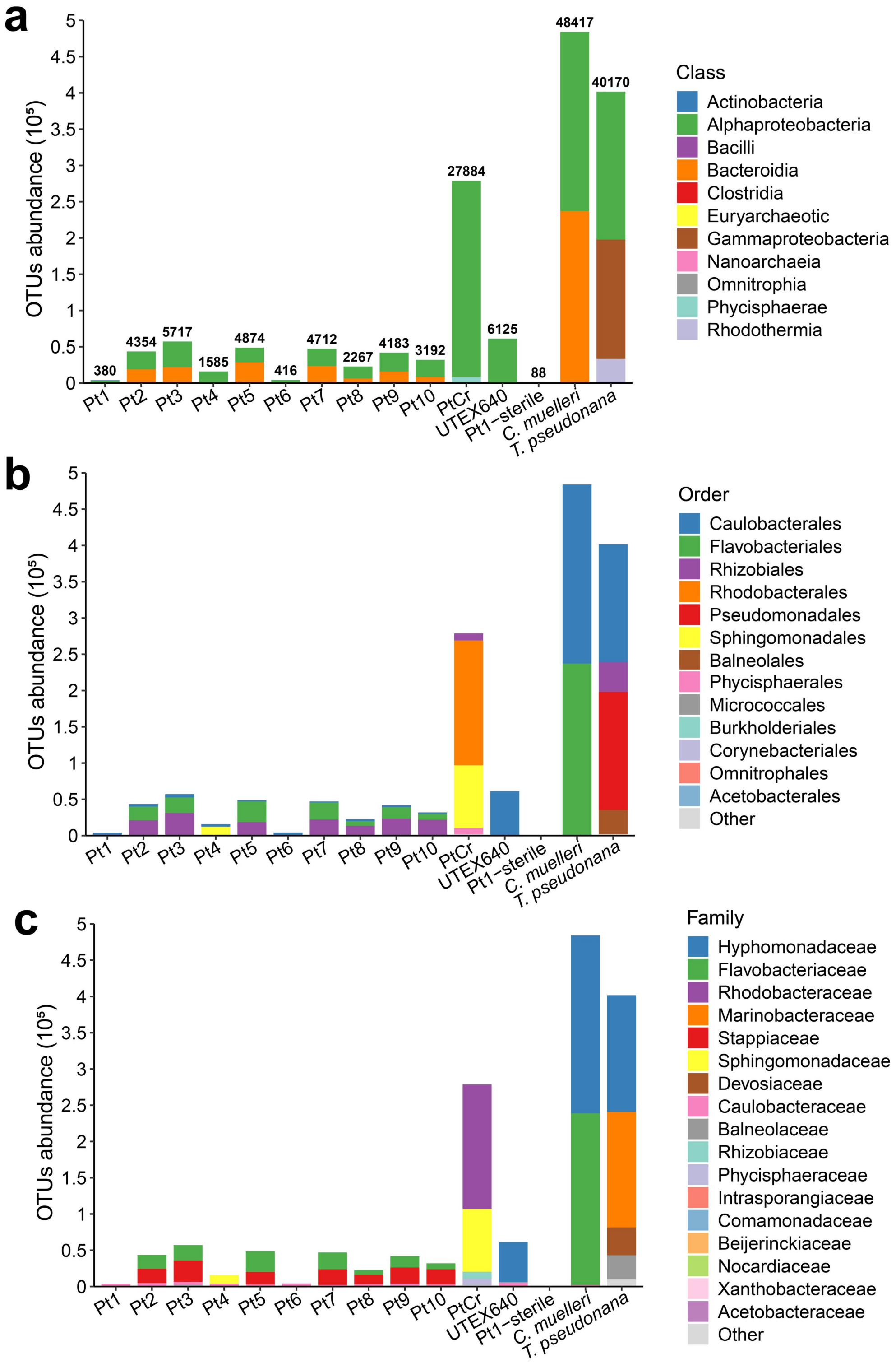

Supplementary Fig. 4 Estimation of operational taxonomic unit (OTU) abundance in associated bacteria of 13 *P. tricornutum* strains together with *C. muelleri* and *T. pseudonana*. Bacterial communities at the class level (**a**) and the four bacterial communities with the highest OTU abundance per sample at order (**b**), and family (**c**) levels were showed. Pt1-sterile, Pt1 with intensified antibiotic treatment. Source data are provided as a Source Data file.

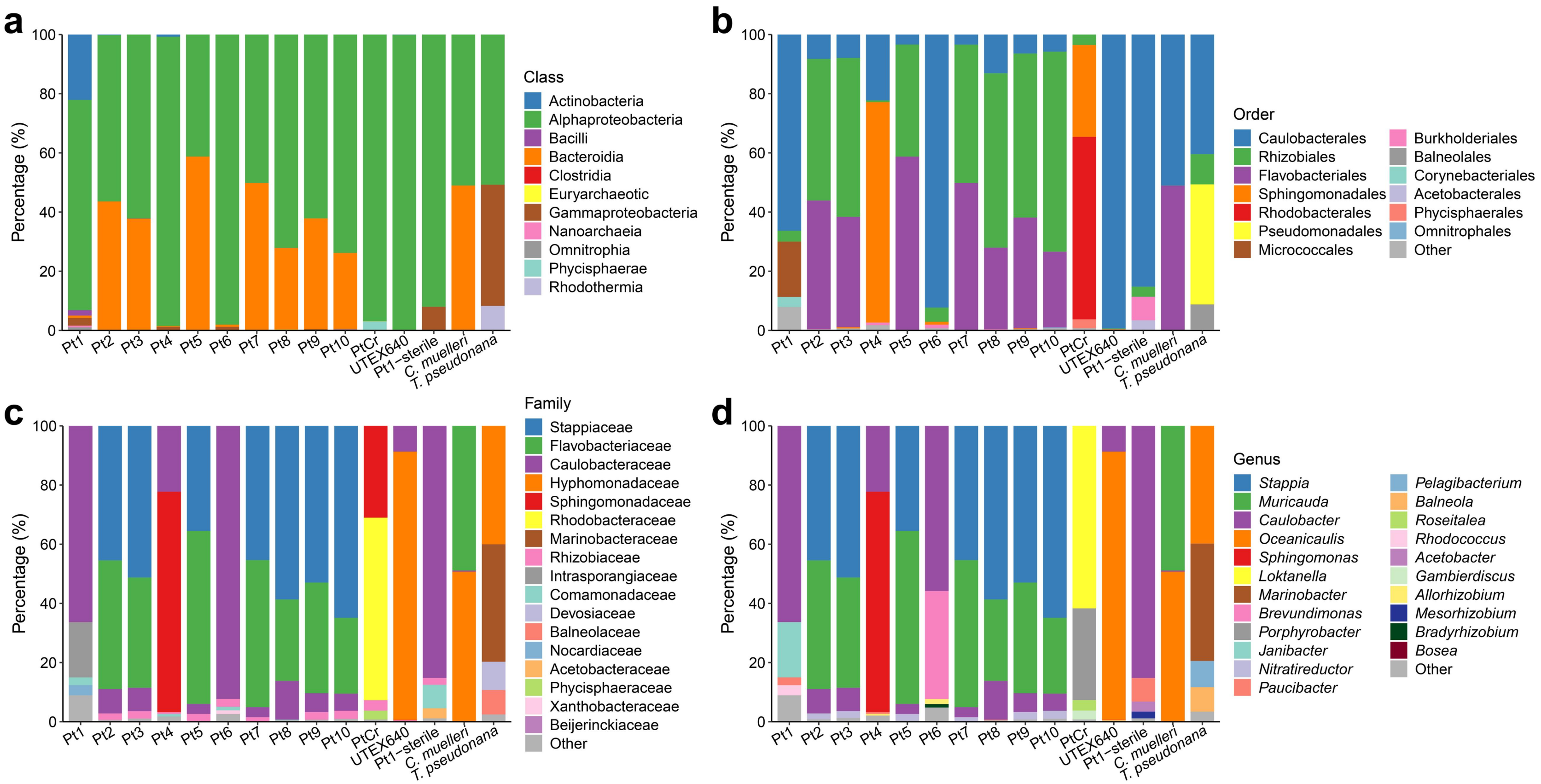

Supplementary Fig. 5 Relative abundance estimation in associated bacteria of 13 *P. tricornutum* strains together with *C. muelleri* and *T. pseudonana*. Bacterial communities at the class level (**a**) and the four bacterial communities with the highest relative abundance per sample at order (**b**), family (**c**), and genus (**d**) levels. Pt1-sterile, Pt1 with intensified antibiotic treatment. Source data are provided as a Source Data file.

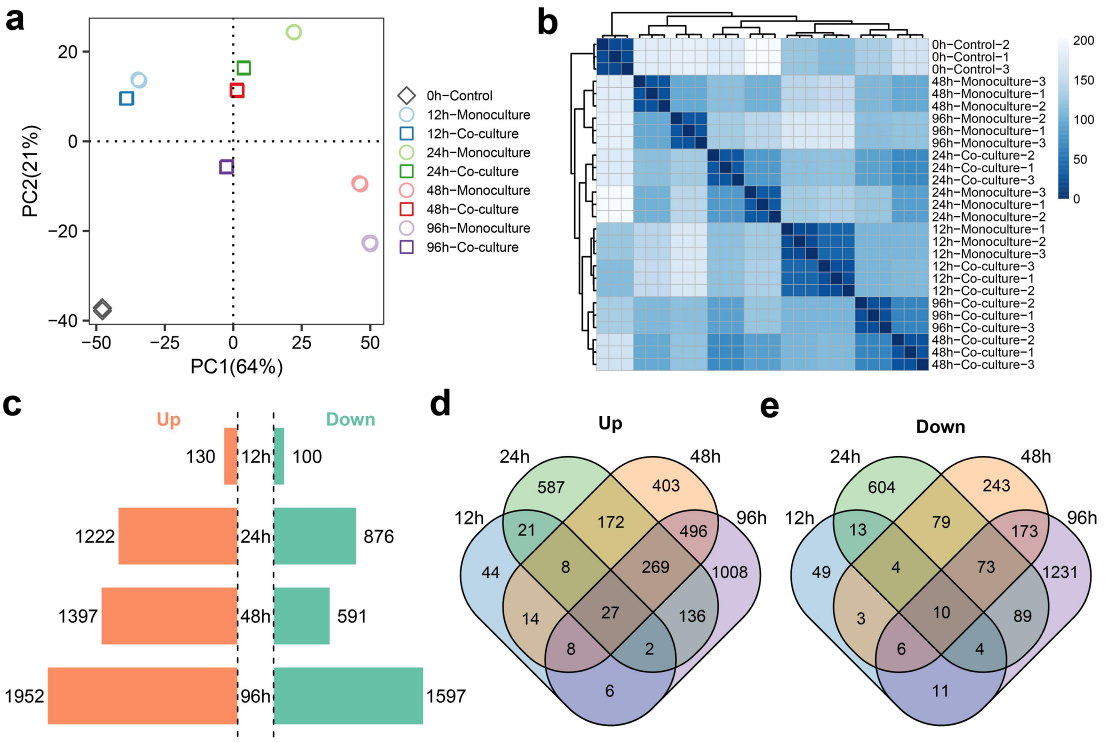

Supplementary Fig. 6 Principal component analysis (**a**) and cluster analysis (**b**) using gene expression levels in each sample. (**c**), Histogram showing the number of up- and down-regulated DEGs in each comparison group. Venn diagram showing the number of shared and unique up- (**d**) and down- (**e**) regulated DEGs in different comparisons. Control, initial control; Monoculture, monoculture of *P. tricornutum*; Co-culture, co-culture of *P. tricornutum* and *L. vestfoldensis*; 0h, 12h, 24h, 48h, and 96h, incubation time. DEGs were defined as genes with $|\log_2(\text{fold change})| > 1$ and q-value < 0.05 (co-culture/algal monoculture, two-sided t-test, 4 degrees of freedom, Benjamini-Hochberg correction, $n = 3$ biological replicates per group) for the same incubation time in *P. tricornutum*. Source data are provided as a Source Data file.

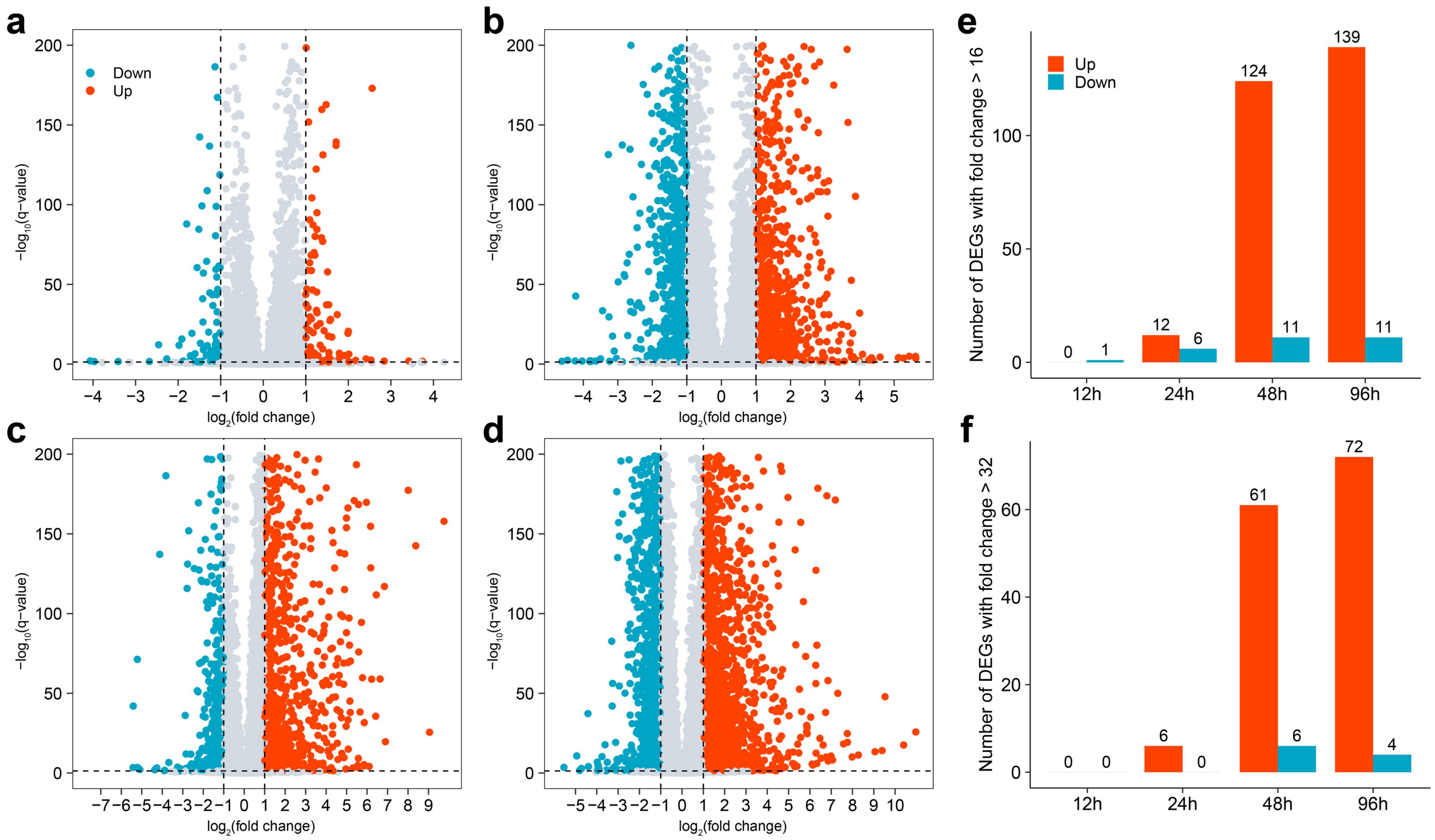

Supplementary Fig. 7 Volcano plots from differential gene expression in pairwise comparisons of cultures at 12 (**a**), 24 (**b**), 48 (**c**), and 96 (**d**) h. (**e**), (**f**), the number of up- or down-regulated DEGs with fold change > 16 and 32 times (co-culture/algal monoculture) at different incubation time, respectively. Source data are provided as a Source Data file.

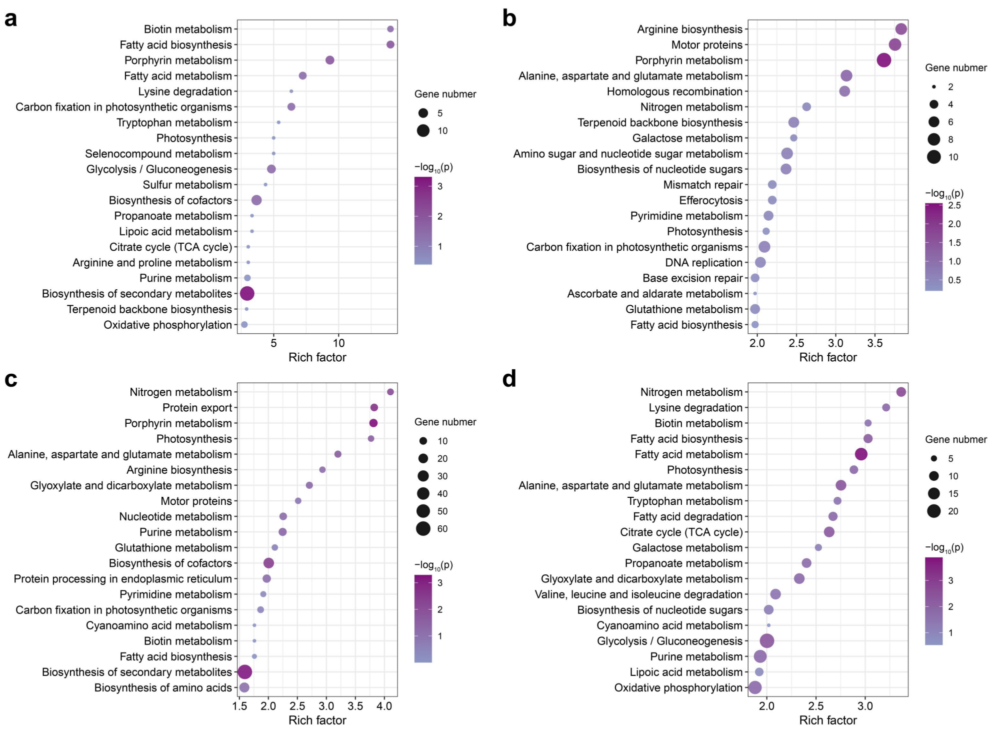

Supplementary Fig. 8 KEGG enrichment analysis of up-regulated DEGs in pairwise comparisons of cultures at 12 (**a**), 24 (**b**), 48 (**c**), and 96 (**d**) h. Source data are provided as a Source Data file.

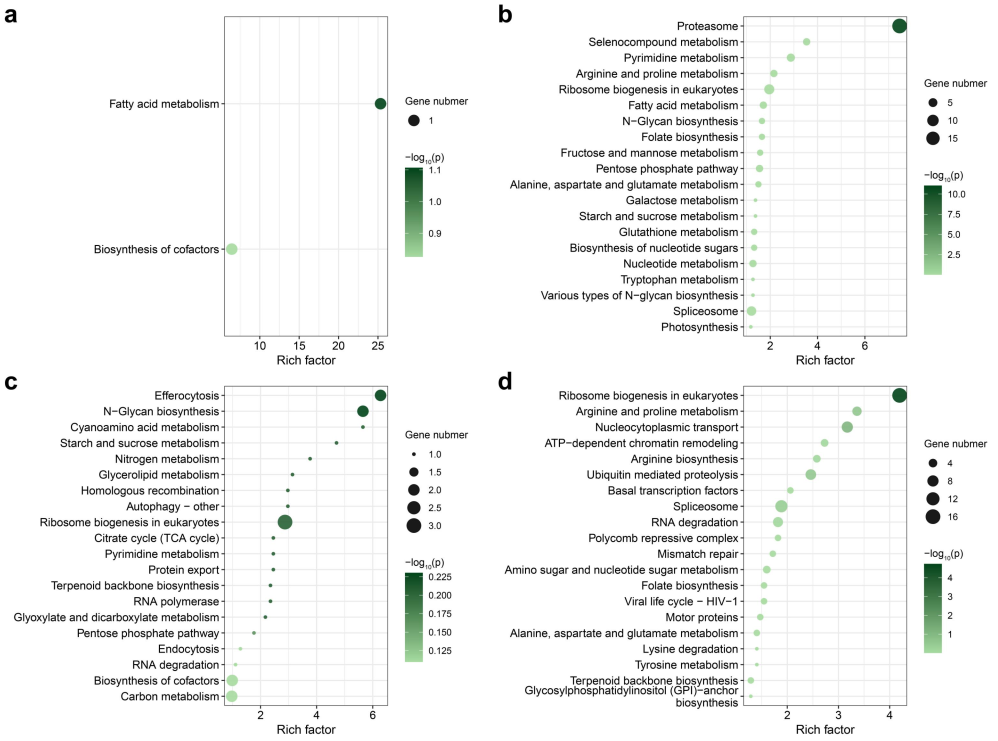

Supplementary Fig. 9 KEGG enrichment analysis of down-regulated DEGs in pairwise comparisons of cultures at 12 (**a**), 24 (**b**), 48 (**c**), and 96 (**d**) h. Source data are provided as a Source Data file.

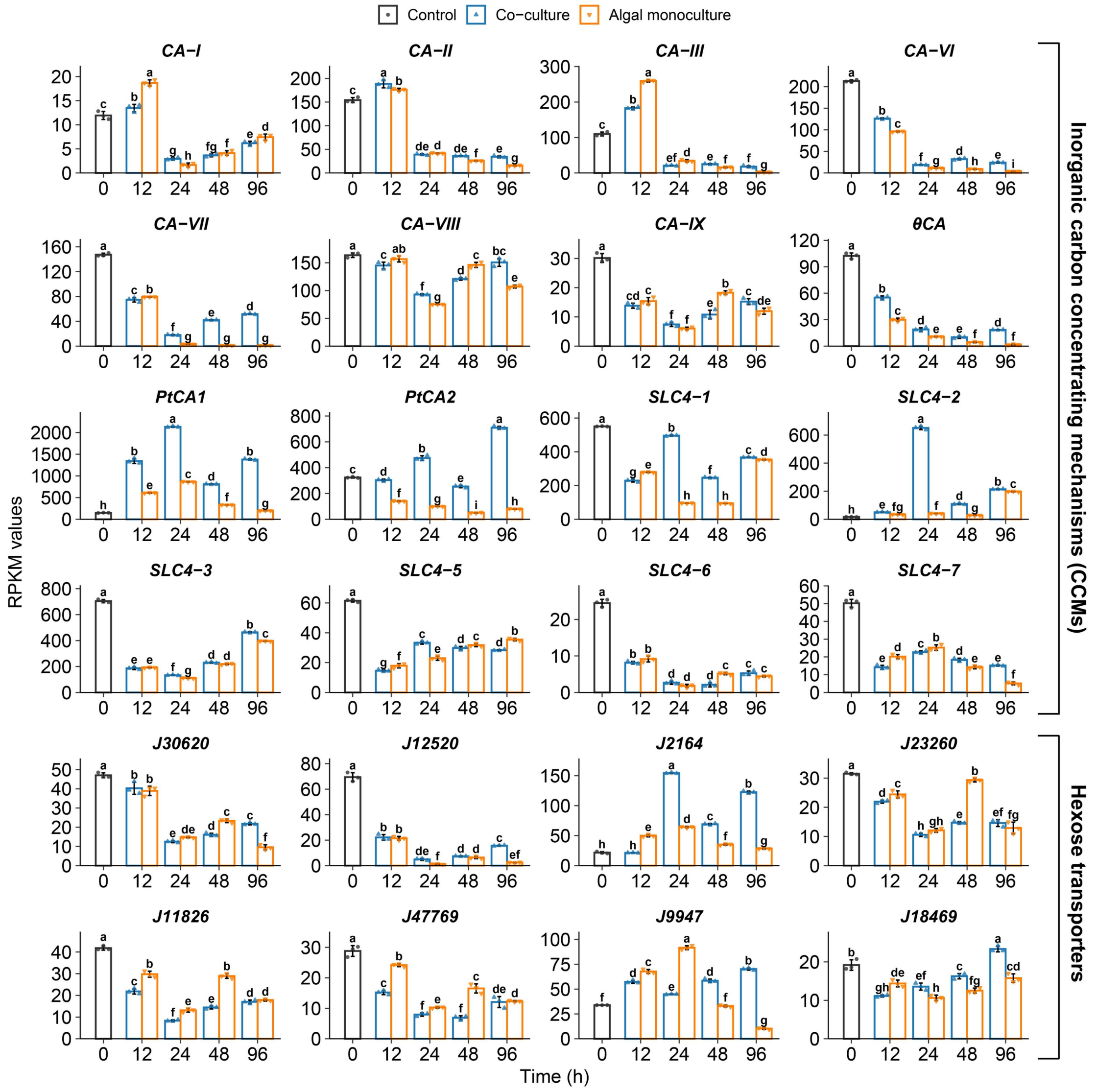

Supplementary Fig. 10 RPKM values of carbonic anhydrase (CA), solute carrier family 4 (SLC4), and hexose transporter genes. The bar plots represent mean ± standard error (n = 3 biological replicates). *CA-III*, *J55029*; *CA-VI*, *J54251*; *CA-VII*, *J42574*; *CA-I*, *J35370*; *CA-II*, *J44526*; *PtCA1*, *J51305*; *PtCA2*, *J45443*; *θCA*, *J43233*; *CA-VIII*, *EG02042*; *CA-IX*, *J36906*; *SLC4-1*, *EG02360*; *SLC4-2*, *Jdraft1806*; *SLC4-3*, *EG02538*; *SLC4-5*, *J54405*; *SLC4-6*, *J43194*; *SLC4-7*, *J45656*. Control, initial control; Co-culture, co-culture of *P. tricornutum* and *L. vestfoldensis*; Algal monoculture, monoculture of *P. tricornutum*. Different letters above the bars indicate statistically significant differences by two-tailed Fisher's LSD test (18 degrees of freedom, $p < 0.05$, Benjamini-Hochberg correction). Source data are provided as a Source Data file.

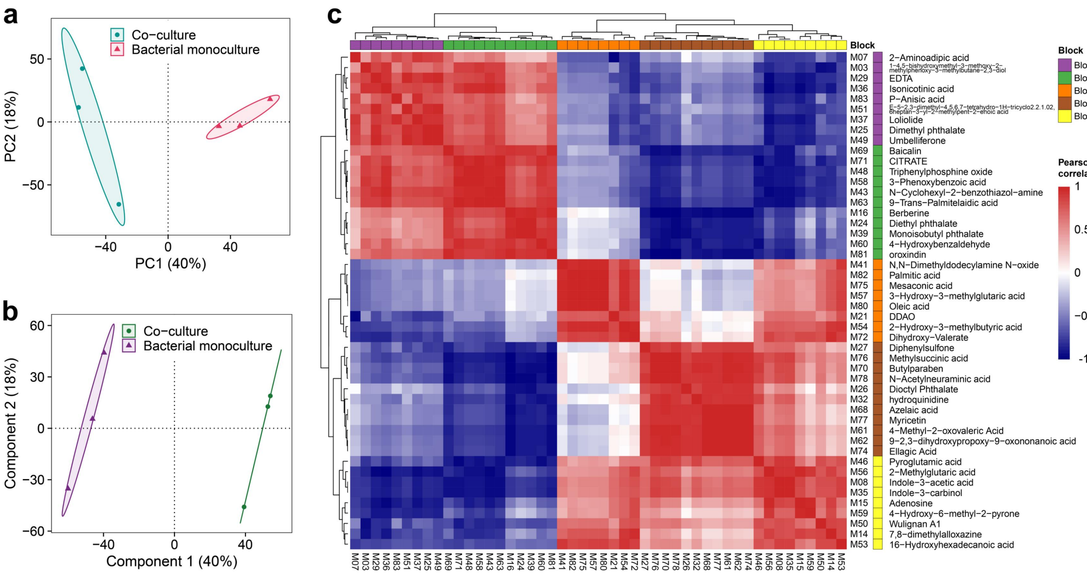

Supplementary Fig. 11 Principal component analysis (**a**) and partial least squares discriminant analysis (**b**) of all characterized peaks in the metabolome. **c**, correlation analysis of 48 out of 84 metabolites from MS2 data (the rest 36 with lower correlation were not shown here). Co-culture, co-culture of *L. vestfoldensis* and *P. tricornutum*; Bacterial monoculture, monoculture of *L. vestfoldensis*. Each group has three biological replicates ($n = 3$). Source data are provided as a Source Data file.

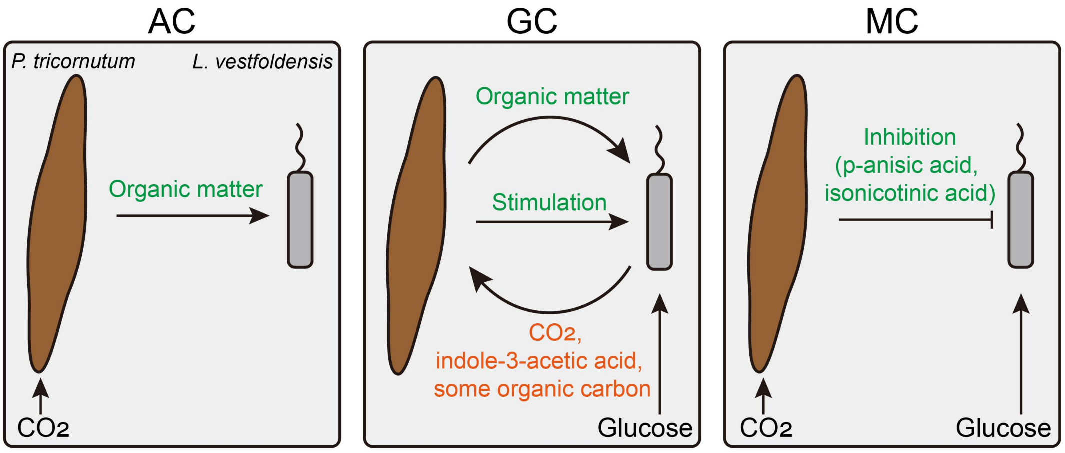

Supplementary Fig. 12 Models of *P. tricornutum* and *L. vestfoldensis* interactions under different carbon sources. AC, using atmospheric CO_2 as the sole carbon source; GC, using 3 g/L glucose as the sole carbon source; MC, using mixed carbon sources (atmospheric CO_2 and 3 g/L glucose). Green and orange font represent metabolites released by *P. tricornutum* and *L. vestfoldensis*, respectively.

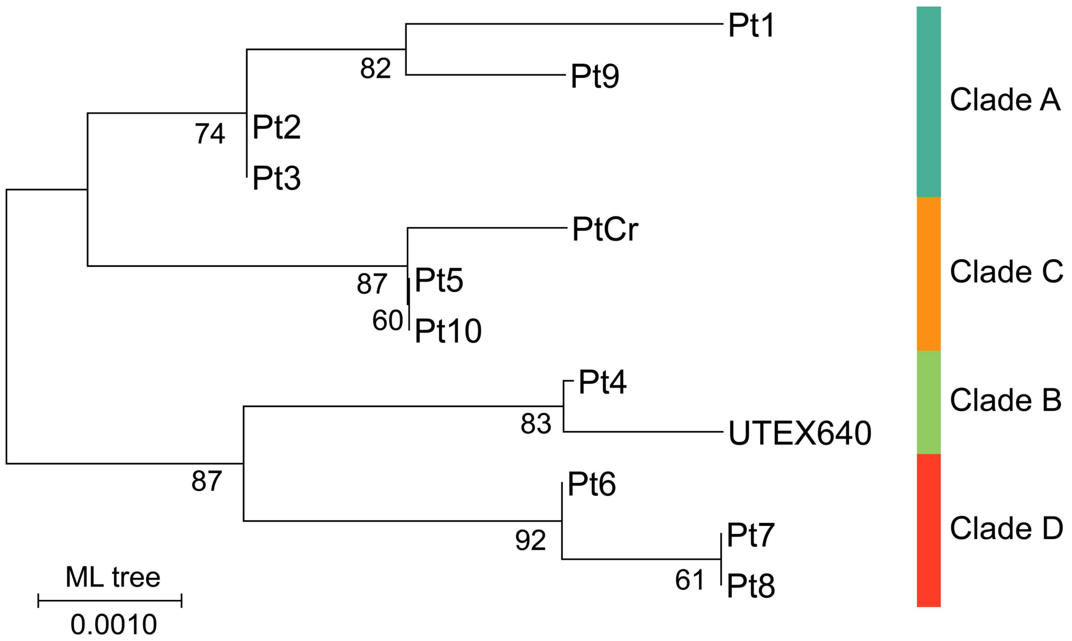

Supplementary Fig. 13 Phylogenetic association of different accessions of *P. tricornutum* based on internal transcribed space (ITS) using a maximum likelihood approach. The four genetic clades (Clade A–D) of *P. tricornutum* have been described in Rastogi et al.[1]

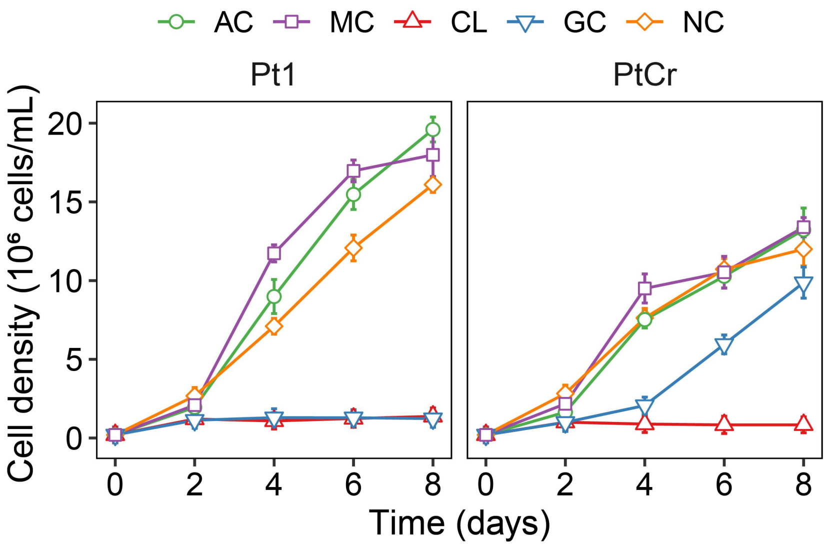

Supplementary Fig. 14 Cell density dynamics of Pt1 and PtCr under different carbon source conditions. The line plots represent mean ± standard error (n = 3 biological replicates). AC, using atmospheric CO_2 as the sole carbon source; MC, using mixed carbon sources (atmospheric CO_2 and 3 g/L glucose); CL, carbon limitation; GC, using 3 g/L glucose as the sole carbon source; NC, addition of 2 mM $NaHCO_3$ under AC. Source data are provided as a Source Data file.

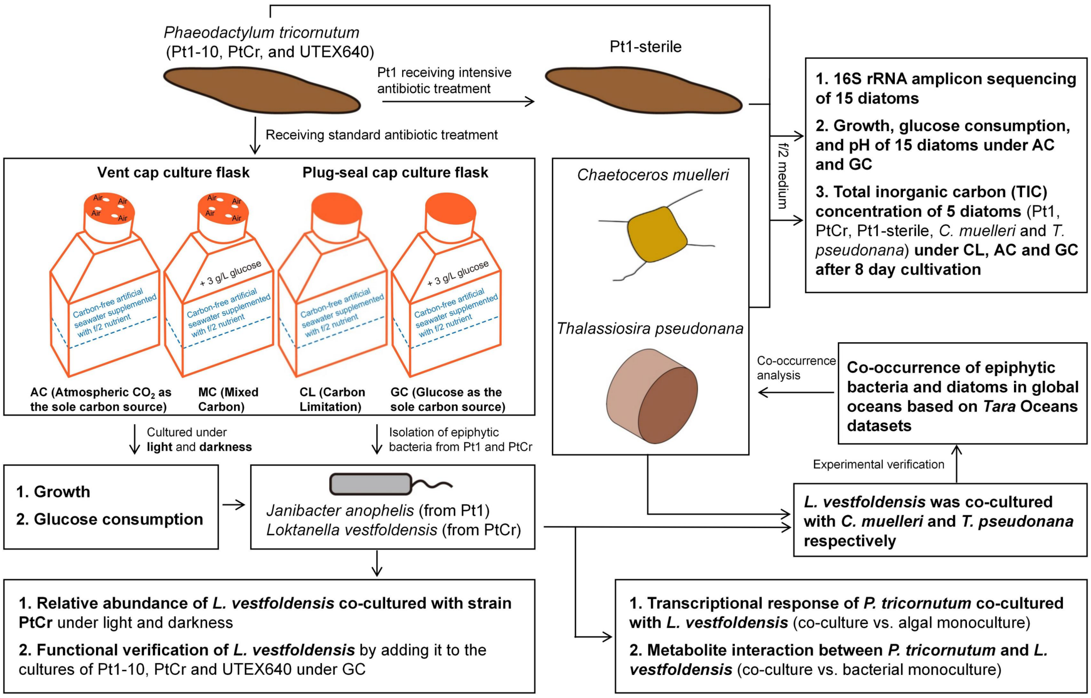

Supplementary Fig. 15 Schematic diagram of the experimental design.

Supplementary Table 1. The number of reads for 16S rNRA amplicon sequencing. Pt1-sterile, Pt1 with intensified antibiotic treatment.

Sample ID	Raw Reads	Clean Reads
Pt1	80125	72640
Pt2	79870	72507
Pt3	76787	69307
Pt4	80060	72624
Pt5	79983	72925
Pt6	80043	72286
Pt7	79944	72633
Pt8	80063	72769
Pt9	79933	71526
Pt10	80247	73046
PtCr	79952	72650
UTEX640	79976	72361
Pt1-sterile	80122	71554
C. muelleri	79997	73285
T. pseudonana	79947	73649

Supplementary Table 2. Identification of epiphytic bacterium of *P. tricornutum.*

Source	Bacterial species	NCBI ID	Identity (%)	Coverage (%)
Pt1	*Janibacter anophelis*	MT225698	99.9	100.0
PtCr	*Loktanella vestfoldensis*	MF289169	99.6	99.0

Supplementary Table 3. Information and mapped rate of transcriptomes in *P. tricornutum.* Control, initial control; Monoculture, monoculture culture of *P. tricornutum*; Co-culture, co-culture of *L. vestfoldensis* and *P. tricornutum.* Each group has three biological replicates ($n = 3$).

Sample	Reads number (M)	Bases number (G)	Q30 (%)	Mapped Rate (%)
0h_Control_1	46.9	7.0	90.4	93.4
0h_Control_2	47.0	7.0	90.7	93.4
0h_Control_3	45.7	6.8	91.2	93.7
12h_Co-culture_1	46.8	7.0	90.0	93.2
12h_Co-culture_2	48.0	7.2	89.9	93.4
12h_Co-culture_3	48.1	7.2	89.4	92.7
12h_Monoculture_1	44.1	6.6	91.1	94.0
12h_Monoculture_2	47.0	7.0	90.9	93.9
12h_Monoculture_3	47.9	7.1	90.9	93.9
24h_Co-culture_1	49.1	7.3	89.9	93.5
24h_Co-culture_2	47.6	7.1	90.4	93.6
24h_Co-culture_3	47.5	7.1	92.4	93.9
24h_Monoculture_1	49.6	7.4	89.9	93.8
24h_Monoculture_2	47.1	7.0	89.8	93.5
24h_Monoculture_3	48.1	7.2	90.2	93.7
48h_Co-culture_1	49.2	7.3	93.3	93.9
48h_Co-culture_2	45.8	6.8	93.7	94.1
48h_Co-culture_3	47.7	7.1	93.2	94.2
48h_Monoculture_1	48.2	7.2	90.2	93.0
48h_Monoculture_2	46.8	7.0	89.8	93.1
48h_Monoculture_3	45.5	6.8	90.1	93.2
96h_Co-culture_1	46.8	7.0	93.1	94.2
96h_Co-culture_2	41.8	6.2	93.5	94.3
96h_Co-culture_3	46.5	6.9	93.5	94.2
96h_Monoculture_1	47.5	7.1	89.5	92.9
96h_Monoculture_2	47.4	7.1	90.5	93.4
96h_Monoculture_3	48.9	7.3	90.1	93.2

Supplementary Table 4. Genes that are consistently up-regulated or down-regulated at four time points in the transcriptome of *P. tricornutum.* Statistical significance was determined by hypergeometric test (co-culture vs. algal monoculture; 4 degrees of freedom, $p < 0.05$, Benjamini-Hochberg correction). Algal monoculture, monoculture culture of *P. tricornutum*; Co-culture, co-culture of *L. vestfoldensis* and *P. tricornutum.* Each group has three biological replicates ($n = 3$).

Gene ID	Name	log2 FC	*p* value	*p* adjust	Function
**Up-regulated**					
Phatr3_EG00198		1.01	0.000	0.000	
Phatr3_EG01033		1.16	0.000	0.000	
Phatr3_J10068	FABI	1.21	0.000	0.000	Enoyl-acp reductase
Phatr3_J10640	HEMF_2	1.97	0.000	0.000	Coproporphyrinogen oxidase Coproporphyrinogenase
Phatr3_J22122	GAPC1	1.49	0.000	0.000	Glyceraldehyde-3-phosphate dehydrogenase
Phatr3_J23850		1.43	0.000	0.000	
Phatr3_J25308	TPI/ GAPC3	1.26	0.000	0.000	Triosephosphate isomerase/ glyceraldehyde-3-phosphate dehydrogenase
Phatr3_J32747	GAPC4	1.40	0.000	0.000	Glyceraldehyde-3-phosphate dehydrogenase
Phatr3_J33530		1.26	0.000	0.000	Methyltransferase domain-containing protein
Phatr3_J33543		1.08	0.000	0.000	PDZ domain-containing protein
Phatr3_J34536	LHCF16	1.26	0.000	0.000	Protein fucoxanthin chlorophyll a/c protein
Phatr3_J36322		1.71	0.000	0.000	Protein kinase domain-containing protein
Phatr3_J37006		1.05	0.004	0.008	Uncharacterized protein
Phatr3_J37652	FABD	1.99	0.000	0.000	Malonyl-CoA:ACP transacylase
Phatr3_J38124		1.14	0.000	0.000	Uncharacterized protein
Phatr3_J40692		1.54	0.000	0.000	Uncharacterized protein
Phatr3_J42282		1.74	0.000	0.000	sulfate adenylyltransferase
Phatr3_J44340		1.53	0.000	0.000	Transmembrane protein
Phatr3_J45443	PTCA2	1.21	0.000	0.000	Carbonic anhydrase
Phatr3_J45690		1.05	0.000	0.000	AAA+ ATPase domain-containing protein
Phatr3_J47006		1.37	0.000	0.000	Uncharacterized protein
Phatr3_J47612		1.80	0.000	0.000	Phytase-like domain-containing protein
Phatr3_J47730		1.34	0.000	0.000	Smr domain-containing protein
Phatr3_J51305	CA	1.23	0.000	0.000	Carbonic anhydrase
Phatr3_J53935	RPE	1.40	0.000	0.000	Ribulose-phosphate 3-epimerase
Phatr3_J54465	ISIP2A	1.40	0.000	0.000	Iron starvation induced protein
Phatr3_Jdraft1828		1.17	0.000	0.000	
**Down-regulated**					
Phatr3_EG00672		-1.07	0.000	0.000	
Phatr3_EG01212		-2.46	0.000	0.000	
Phatr3_EG01387		-1.18	0.002	0.004	
Phatr3_EG02228		-1.19	0.000	0.000	Mercuric reductase
Phatr3_J15126		-2.01	0.000	0.000	DUF202 domain-containing protein
Phatr3_J22315		-1.42	0.000	0.000	Mitochondrial carrier protein
Phatr3_J45819		-1.00	0.000	0.000	DUF6824 domain-containing protein
Phatr3_J48172		-1.75	0.016	0.030	Uncharacterized protein
Phatr3_J50495		-1.09	0.000	0.000	Uncharacterized protein
Phatr3_J54574		-1.41	0.000	0.000	Kinesin light chain

Supplementary Table 5. The predicted location of 8 hexose transporters by DeepLoc-2.1.

**Gene id**	**Description**	**DeepLoc-2.1 (Predicted location, https://services.healthtech.dtu.dk/services/DeepLoc-2.1/)**
Phatr3_J30620	Hexose transporter 1	Lysosome/Vacuole
Phatr3_J12520	Hexose transporter 1	Lysosome/Vacuole
Phatr3_J2164	Hexose transporter 1	Lysosome/Vacuole | Golgi apparatus
Phatr3_J23260	Hexose transporter 1	Lysosome/Vacuole
Phatr3_J11826	Hexose transporter 1	Lysosome/Vacuole | Golgi apparatus
Phatr3_J47769	Hexose transporter 1	Lysosome/Vacuole
Phatr3_J9947	Hexose transporter 1	Cell membrane | Lysosome/Vacuole
Phatr3_J18469	Hexose transporter	Lysosome/Vacuole

Supplementary Reference

1. Rastogi, A. et al. A genomics approach reveals the global genetic polymorphism, structure, and functional diversity of ten accessions of the marine model diatom *Phaeodactylum tricornutum*. *ISME J.* **14**, 347–363 (2020).
