# Supplementary material for: Interactions with bacteria shape diatom adaptation to carbon concentration changes
Source: Nat Commun. 2025 Dec 27;17:1289. doi: 10.1038/s41467-025-68050-3 (PMC12868695; doi:10.1038/s41467-025-68050-3)
Supplement: Supplementary file 2 — Description of Additional Supplementary Information [file 41467_2025_68050_MOESM2_ESM.pdf]

Description of Additional Supplementary Files

File Name: Supplementary Data 1
Description: Estimation of operational taxonomic unit (OTU) abundance in associated bacteria of 13 *P. tricornutum* strains together with *C. muelleri* and *T. pseudonana*. Pt1-sterile, Pt1 with intensified antibiotic treatment.

File Name: Supplementary Data 2
Description: Distribution and OTU abundance of diatoms and epiphytic bacterium of *P. tricornutum* in the *Tara* Oceans Database. The data file link is: https://www.ebi.ac.uk/biostudies/files/S-BSST297/OM-RGC_v2_taxonomic_profiles.tar.gz.

File Name: Supplementary Data 3
Description: Expression levels of reads counts in the transcriptome of *P. tricornutum*. Control, initial control; Monoculture, monoculture of *P. tricornutum*; Co-culture, co-culture of *P. tricornutum* and *L. vestfoldensis*. 0, 12, 24, 48, and 96h, incubation time. Each group has three biological replicates (n = 3).

File Name: Supplementary Data 4
Description: Expression levels of RPKM in the transcriptome of *P. tricornutum*. Control, initial control; Monoculture, monoculture of *P. tricornutum*; Co-culture, co-culture of *P. tricornutum* and *L. vestfoldensis*. 0, 12, 24, 48, and 96h, incubation time. Each group has three biological replicates (n = 3).

File Name: Supplementary Data 5
Description: All information of feature peaks and peak intensity in the metabolomics. Co-culture, co-culture of *L. vestfoldensis* and *P. tricornutum*; Monoculture, monoculture of *L. vestfoldensis*. Each group has three biological replicates (n = 3).

File Name: Supplementary Data 6
Description: MS2 annotation information for feature peaks and peak intensity in the metabolomics. Co-culture, co-culture of *L. vestfoldensis* and *P. tricornutum*; Monoculture, monoculture of *L. vestfoldensis*. Statistical significance was determined by two-tailed unpaired t-test (Co-culture vs. Bacterial monoculture; 4 degrees of freedom, $p < 0.05$, Benjamini-Hochberg correction, n = 3 biological replicates per group).
