# Supplementary material for: Interactions with bacteria shape diatom adaptation to carbon concentration changes
Source: Nat Commun. 2025 Dec 27;17:1289. doi: 10.1038/s41467-025-68050-3 (PMC12868695; doi:10.1038/s41467-025-68050-3)
Supplement: Supplementary file 4 — Reporting Summary [file 41467_2025_68050_MOESM4_ESM.pdf]

nature portfolio

Corresponding author(s): Hanhua Hu

Last updated by author(s): Nov 17, 2025

Reporting Summary

Nature Portfolio wishes to improve the reproducibility of the work that we publish. This form provides structure for consistency and transparency in reporting. For further information on Nature Portfolio policies, see our Editorial Policies and the Editorial Policy Checklist.

Statistics

For all statistical analyses, confirm that the following items are present in the figure legend, table legend, main text, or Methods section.

n/a	Confirmed	
☐	☒	The exact sample size (n) for each experimental group/condition, given as a discrete number and unit of measurement
☐	☒	A statement on whether measurements were taken from distinct samples or whether the same sample was measured repeatedly
☐	☒	The statistical test(s) used AND whether they are one- or two-sided<br>*Only common tests should be described solely by name; describe more complex techniques in the Methods section.*
☒	☐	A description of all covariates tested
☒	☐	A description of any assumptions or corrections, such as tests of normality and adjustment for multiple comparisons
☐	☒	A full description of the statistical parameters including central tendency (e.g. means) or other basic estimates (e.g. regression coefficient) AND variation (e.g. standard deviation) or associated estimates of uncertainty (e.g. confidence intervals)
☐	☒	For null hypothesis testing, the test statistic (e.g. F, t, r) with confidence intervals, effect sizes, degrees of freedom and P value noted<br>*Give P values as exact values whenever suitable.*
☒	☐	For Bayesian analysis, information on the choice of priors and Markov chain Monte Carlo settings
☒	☐	For hierarchical and complex designs, identification of the appropriate level for tests and full reporting of outcomes
☒	☐	Estimates of effect sizes (e.g. Cohen's d, Pearson's r), indicating how they were calculated

Our web collection on statistics for biologists contains articles on many of the points above.

Software and code

Policy information about availability of computer code

Data collection

1. RNA-seq was performed with Illumina HiSeq 2000 at Annoroad Gene Technology (Peking, China). 2. Quantitative real time PCR (qPCR) was performed on a Bio-Rad CFX96 real-time PCR system (Bio-RAD, USA) following the MIQE guidelines. 3. Metabolite profiling was performed with Ultimate 3000 UHPLC system coupled to a Q Exactive high-resolution mass spectrometry (Thermo Fisher Scientific, USA) at Anachro Technologies Inc. (Wuhan, China).
2. The V3-V4 region of 16S rRNA gene of epiphytic bacteria were amplified using universal primer. The PCR amplicons were purified with Agencourt AMPure XP Beads (Beckman Coulter, USA) and quantified using the Qubit 4.0 Fluorometer (Thermo Fisher Scientific, USA). Qualified amplicons were used to construct libraries and sequenced using Illumina novaseq 6000 (Illumina, USA) with 150 bp paired-end reads.
3. Metabolite profiling was performed using an Ultimate 3000 UHPLC system (Thermo Fisher Scientific, USA) coupled to a Q Exactive Orbitrap mass spectrometry (Thermo Fisher Scientific, USA) equipped with a Waters ACQUITY UPLC HSS T3 column (Waters, USA; 100 mm × 2.1 mm × 1.8 µm). Both positive and negative ionization modes were employed for comprehensive metabolite detection. Mobile phases consisted of: (1) positive mode - 0.1% formic acid in water (A) and 0.1% formic acid in methanol (B); (2) negative mode - 10 mM ammonium formate in water (A) and 10 mM ammonium formate in 95% methanol (B). The gradient program was: 0-1 min, 10% B; 1-13 min, linear increase to 98% B; 13-18 min, 98% B; 18-18.5 min, linear decrease to 10% B; 18.5-20 min, 10% B. The injection volume for each sample was 2 µL, and methanol was used as a blank sample to deduct baseline features from the mobile phase. The mass spectrometer was operated in a data-dependent acquisition (DDA) mode with positive and negative ions scanned in separate runs. The acquisition cycle consisted of a full MS scan at a resolution of 70,000, followed by data-dependent MS2 (dd-MS2) scans of the most intense precursors at a resolution of 17,500. Electrospray ionization (ESI) source parameters were set as follows: spray voltages, 3.8 kV for positive mode and 3.2 kV for negative mode; capillary temperature, 300 °C; sheath gas flow rate, 0.3 mL/min; and nebulizer temperature, 350 °C. Samples were maintained at 4 °C in the autosampler during analysis.

Data analysis	1. Raw RNA-Seq reads were filtered using Fastp (version 0.12.4) to remove adapters and low-quality reads. Clean reads were mapped to the reference genome of P. tricornutum using Hisat2 (version 2.1.0). FeatureCount (version 2.0.1) was used to obtain read counts for each gene and manually calculate reads per kilobase of transcript per million mapped reads (RPKM). Gene expression was normalized using the R package DESeq2 (version 1.28.1). PCA and cluster analysis were performed on normalized data. DEGs were identified using stringent thresholds (|log2FC| > 1, q-value < 0.05) comparing co-culture and algal monoculture conditions. The number of DEGs was counted, and pathway enrichment analysis was performed for DEGs based on the Kyoto Encyclopedia of Genes and Genomes (KEGG) using the R package clusterProfiler (version 3.16.1).<br>2. Raw data of 16S rRNA amplicon sequencing was primarily filtered to abtain clean reads by Trimmomatic (version 0.33) and Cutadapt (version 1.9.1). Clean reads were assembled and and followed by chimera removal using DADA2. Sequences with similarity ≥ 97% were clustered into the same operational taxonomic unit (OTU) by VSEARCH (version 2.26.1), and the OTUs with reabundace < 0.005% and abundace < 5 were filtered. Use BLAST to remove OTUs with similar sequences to the mitochondria and chloroplast genomes of P. tricornutum and C. muelleri, with a filtering condition of similarity > 90%, e-value < 10^-5. Taxonomy annotation of the OTUs was performed based on the Naive Bayes classifier in QIIME2 using the SILVA database (release 138) with a confidence threshold of 70%.<br>3. Use MS-DIAL software (version 4.70) to perform blank deduction (peak intensity [Sample/Blank] >= 3), feature peak screening (at least 2 feature peaks appearing in repeated samples), and normalization (lowess method) on the merged data of positive and negative ion modes. A comprehensive feature matrix was generated, containing metabolite identifiers, RT, mass-to-charge ratio (m/z), ion patterns, and peak intensities. Metabolite annotation was achieved by matching experimental RT and m/z values against the MassBank database with the following parameters: RT tolerance = ± 0.1 min, MS1 mass tolerance = 0.005 Da, MS2 mass tolerance = 0.0025 Da, and MS2 spectral match score > 0.7. Missing values were imputed using 20% of the minimum value within each experimental group. The peak intensities were subjected to Pareto scaling and log10 transformation to minimize magnitude-related artifacts. Multivariate statistical analyses, including PCA, PLS-DA, and hierarchical clustering, were performed using R and R package mixOmics (version 6.24.0) to evaluate intergroup variability, metabolite correlations, and differential features. Differentially abundant metabolites under co-culture and bacterial monoculture conditions were identified using Student's t-test, with stringent thresholds of |log2FC| > 1 and a q-value < 0.05 (Benjamini-Hochberg adjusted p-value).<br>4. To investigate the distribution of symbiotic bacteria and diatoms in the global ocean, the abundance of diatoms (Bacillariophyceae), Janibacter, and Loktanella and the bacterial-algal co-occurrence were analyzed, based on species diversity data from 180 macrogenomes in the Tara Oceans database (https://www.ebi.ac.uk/biostudies/files/S-BSST297/OM-RGC_v2_taxonomic_profiles.tar.gz).

For manuscripts utilizing custom algorithms or software that are central to the research but not yet described in published literature, software must be made available to editors and reviewers. We strongly encourage code deposition in a community repository (e.g. GitHub). See the Nature Portfolio guidelines for submitting code & software for further information.

Data

Policy information about availability of data

All manuscripts must include a data availability statement. This statement should provide the following information, where applicable:

- Accession codes, unique identifiers, or web links for publicly available datasets
- A description of any restrictions on data availability
- For clinical datasets or third party data, please ensure that the statement adheres to our policy

The raw data of RNA-Seq and 16S rRNA amplicon sequencing have been deposited in the Sequence Read Archive database under accession number PRJNA1276728 and PRJNA1330494. The Sanger sequencing data of 16S rRNA have been deposited in the Genbank database under accession numbers PX410800 (Janibacter anophelis) and PX410801 (Loktanella vestfoldensis). The metabolomic data have been deposited on Metabolomics Workbench under Study ID ST004351 (http://dx.doi.org/10.21228/M8WR9Z). The code used to produce the results are available at Figshare (https://doi.org/10.6084/m9.figshare.30178000). Source Data are provided with this Paper.

Research involving human participants, their data, or biological material

Policy information about studies with human participants or human data. See also policy information about sex, gender (identity/presentation), and sexual orientation and race, ethnicity and racism.

Reporting on sex and gender	This study did not involve human research participants.
Reporting on race, ethnicity, or other socially relevant groupings	This study did not involve human research participants.
Population characteristics	This study did not involve human research participants.
Recruitment	This study did not involve human research participants.
Ethics oversight	This study did not involve human research participants.

Note that full information on the approval of the study protocol must also be provided in the manuscript.

Field-specific reporting

Please select the one below that is the best fit for your research. If you are not sure, read the appropriate sections before making your selection.

[x] Life sciences [ ] Behavioural & social sciences [ ] Ecological, evolutionary & environmental sciences

For a reference copy of the document with all sections, see nature.com/documents/nr-reporting-summary-flat.pdf

Life sciences study design

All studies must disclose on these points even when the disclosure is negative.

Sample size	The sample sizes and results of statistical analysis were described in the methods section. Sample sizes were based on similar studies published previously and were sufficient to show statistical differences between the experimental group and the control group.
Data exclusions	No data was excluded from the analysis.
Replication	All the data were based on at least 3 independent biology replicates to ensure the reliability of the results, and the number of replicates is indicated in the corresponding methods section.
Randomization	Samples were selected randomly.
Blinding	Noblinding was done as none of the experiments described in this study involve group allocation during data collection or analyses.

Reporting for specific materials, systems and methods

We require information from authors about some types of materials, experimental systems and methods used in many studies. Here, indicate whether each material, system or method listed is relevant to your study. If you are not sure if a list item applies to your research, read the appropriate section before selecting a response.

Materials & experimental systems

n/a	Involved in the study	
☒	☐	Antibodies
☒	☐	Eukaryotic cell lines
☒	☐	Palaeontology and archaeology
☒	☐	Animals and other organisms
☒	☐	Clinical data
☒	☐	Dual use research of concern
☒	☐	Plants

Methods

n/a	Involved in the study	
☒	☐	ChIP-seq
☒	☐	Flow cytometry
☒	☐	MRI-based neuroimaging

Plants

Seed stocks	The two main Phaeodactylum tricornutum strains were obtained from Provasoli-Guillard National Center for Culture of Marine Phytoplankton (Pt1 = CCMP632) and Institute of Oceanography of Chinese Academy of Sciences (PtCr = CCMM 2004).
Novel plant genotypes	N/A
Authentication	N/A
